# Ultrasonic microbubble VEGF gene delivery improves angiogenesis of senescent endothelial progenitor cells

**DOI:** 10.1038/s41598-021-92754-3

**Published:** 2021-06-29

**Authors:** Yi-Nan Lee, Yih-Jer Wu, Hsin-I. Lee, Hsueh-Hsiao Wang, Chiung-Yin Chang, Ting-Yi Tien, Chao-Feng Lin, Cheng-Huang Su, Hung-I. Yeh

**Affiliations:** 1 Cardiovascular Center, Departments of Medical Research, MacKay Memorial Hospital, No. 92, Sec. 2, Zhongshan N. Rd., Taipei City, 10449 Taiwan; 2 Mackay Medical College, No.46, Sec. 3, Zhongzheng Rd. Sanzhi Dist. 252, New Taipei City, Taiwan

**Keywords:** Cell biology, Stem cells, Cardiology

## Abstract

The therapeutic effects of ultrasonic microbubble transfection (UMT)-based vascular endothelial growth factor 165 (VEGF165) gene delivery on young and senescent endothelial progenitor cells (EPCs) were investigated. By UMT, plasmid DNA (pDNA) can be delivered into both young EPCs and senescent EPCs. In the UMT groups, higher pDNA-derived protein expression was found in senescent EPCs than in young EPCs. Consistent with this finding, a higher intracellular level of pDNA copy number was detected in senescent EPCs, with a peak at the 2-h time point post UMT. Ultrasonic microbubble delivery with or without VEGF improved the angiogenic properties, including the proliferation and/or migration activities, of senescent EPCs. Supernatants from young and senescent EPCs subjected to UMT-mediated VEGF transfection enhanced the proliferation and migration of human aortic endothelial cells (HAECs), and the supernatant of senescent EPCs enhanced proliferation more strongly than the supernatant from young EPCs. In the UMT groups, the stronger enhancing effect of the supernatant from senescent cells on HAEC proliferation was consistent with the higher intracellular VEGF pDNA copy number and level of protein production per cell in the supernatant from senescent cells in comparison to the supernatant from young EPCs. Given that limitations for cell therapies are the inadequate number of transplanted cells and/or insufficient cell angiogenesis, these findings provide a foundation for enhancing the therapeutic angiogenic effect of cell therapy with senescent EPCs in ischaemic cardiovascular diseases.

## Introduction

Bone marrow (BM)-derived circulating endothelial progenitor cells (EPCs) have gained attention as a new vascular research target since 1997^1^. EPCs contribute to adult neovascularization by physically incorporating into and, through paracrine mechanisms, promoting the growth of vessels^1,2^. These properties of EPCs have been applied in clinical trials for therapeutic angiogenesis in ischaemic tissues and organs^3–10^.

Ageing is associated with deficient blood levels of growth factors and cytokines, which affects tissues and cells^11^. Cellular senescence or cellular ageing, is defined as a diminished replicative capacity and altered functionality within the cell^12–15^. During ageing, cells, including EPCs, undergo substantial reductions in number and activity^13–17^. Some studies have shown that ageing or senescence constitutes a potential limitation to the neovascularization ability of EPCs in the repair of tissue ischaemia. For example, implantation of BM-derived mononuclear cells (BMCs) into ischaemic hindlimbs was shown to enhance tissue perfusion in young (8-week-old) mice; however, the effect was markedly attenuated when BMCs derived from 18-month-old animals were used^18^. Chronic treatment with BM-derived EPCs from young mice with apolipoprotein E (apoE) deficiency prevented the progression of atherosclerosis in apoE-deficient recipients, and the effectiveness of this therapy was similarly attenuated when the donor mice were older^19^. Additionally, the functional capacities of EPCs from older individuals appeared impaired in proliferation, migration and survival, as shown by in vitro examination^20^. These results strongly indicate that ageing is an important determinant of EPC function and support the hypothesis that alterations in EPC function during the aging process lead to the deterioration of cardiovascular repair mechanisms in the aged host.

Virus-based gene delivery is efficient, but safety factors related to inflammation, immunogenicity, and insertion mutations have historically limited its clinical application in humans. Ultrasound-mediated gene delivery, based on the excellent safety record of ultrasound techniques in clinical imaging, has been shown to be a feasible, non-invasive method for nonviral gene delivery^21,22^. Ultrasound (US) exposure with the biological effect of inertial cavitation^23,24^ can induce transient pore formation in cell membranes (i.e., sonoporation)^25–28^, allowing the entry of molecules such as plasmid DNA (pDNA)^29^ into cells. Microbubbles containing gas have been shown to enhance the effect of ultrasound-assisted in vitro and in vivo gene delivery (i.e., ultrasonic microbubble transfection (UMT))^21,30^.

Furthermore, vascular endothelial growth factor (VEGF) proteins, including VEGF165, which has the strongest biological effects^31^, are potent vascular endothelial cell mitogenic/angiogenic factors and can act directly on vascular endothelial cells in a specific manner to trigger angiogenesis^31–33^. According to our previous optimization experiments in endothelial lineage cells, including EPCs and human aortic endothelial cells (HAECs), UMT performed with a list of optimized US parameters was proven to successfully deliver both luciferase reporter pDNA and functional VEGF165 pDNA into young cells^29,34^.

Although cell therapy with EPCs derived from young (nonageing) individuals has been shown to be beneficial in ischaemia-related cardiovascular diseases, a major limitation for such therapies is the inadequate number of transplanted cells and/or insufficient angiogenic activity of the cells^35^. Although in vitro expansion of the cells by serial passaging increases the cell number, their angiogenic function gradually declines due to the ageing process. Therefore, to investigate whether ageing EPCs can be a feasible cell source for cell therapy and understand the potential clinical implications of this approach, the present study was conducted to assess the effect of UMT-based delivery of the growth factor gene VEGF165 via a time course analysis of intracellular pDNA delivery and to investigate alterations in angiogenic function between young and senescent porcine EPCs.

## Results

### Evaluation and definition of young and senescent EPCs

**Figure 1:**
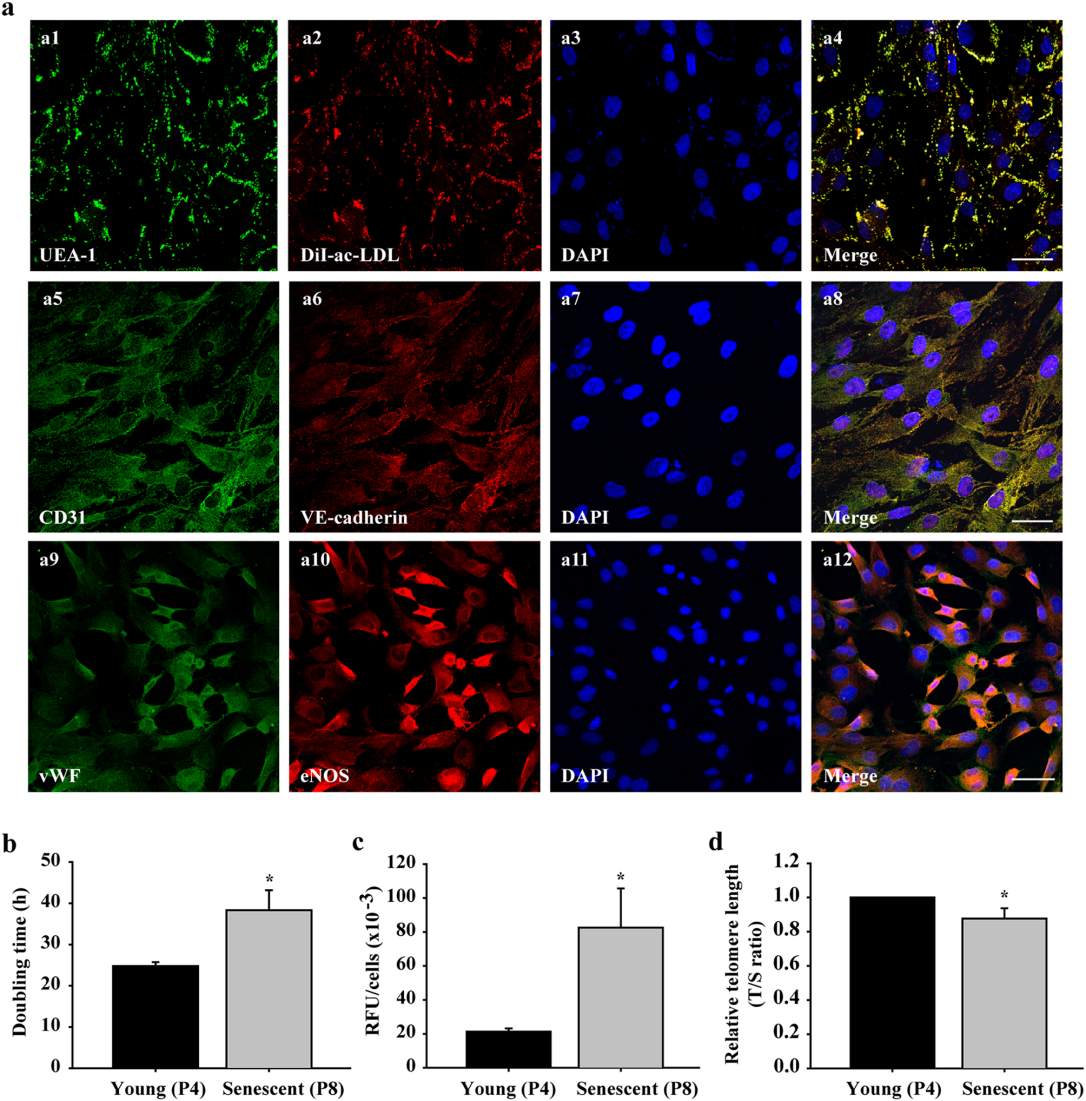
Characterization of EPCs and comparison of young (passage 4, P4)/senescent (passage 8, P8) porcine EPCs. Subconfluent EPCs were fixed with 4% paraformaldehyde (PFA) and stained with the indicated antibodies against UEA-1 (green a1). EPCs were incubated with DiI-labelled ac-LDL (red) for 4 h before PFA fixation (a2). Cell nuclei were visualized by DAPI staining (blue, a3, a7 and a11). Images were then merged (a4, a8 and a12). Cells were stained with anti-CD31 and anti-VE-cadherin (a5 and a6) antibodies. Panels a9 and a10 show cells stained with anti-vWF and anti-eNOS antibodies. Images were acquired by confocal microscopy with a 40 × (oil, na 1.30) magnification objective (SP8, Leica, Germany). Scale bar: 50 μm. P4 (young group) and P8 (senescent group) cells were evaluated for doubling time using a cell counting kit-8 (CCK-8; **b**), for acidic *β*-galactosidase activity using acidic *β*-galactosidase staining (**c**), and for telomere length using real-time PCR (**d**). The telomere (T) repeat copy number was normalized to the single-copy (S) gene 36B4 copy number. The T/S ratio of the control group was set as 1. n = 6 for each bar. *P < 0.05 vs. the young (P4) group. h = hours. RFU = relative fluorescence units.

A comprehensive phenotypic identification of porcine late EPCs was confirmed by the DiI-ac-LDL uptake, UEA-1 lectin binding, and expression of endothelial cell markers (Fig. 1a1–a12).

Senescence-related activities of EPCs after in vitro serial passaging were evaluated using the cell number doubling time, *β*-galactosidase activity, and telomere length. The results showed that the doubling time of the P8 EPCs was markedly longer than that of the P4 cells (39.07 ± 3.18 vs. 25.51 ± 1.13 h, *P* < 0.05, Fig. 1b). *β*-galactosidase activity was enhanced significantly in the P8 EPCs (82.55 × 10^3^ ± 23.10 × 10^3^ relative fluorescence units (RFU)) compared to the P4 cells (21.29 × 10^3^ ± 1.88 × 10^3^ RFU; *P* < 0.05, Fig. 1c). The telomere length of the P8 EPCs was shorter than that of the P4 cells (*P* < 0.05, Fig. 1d). Based on the above findings, the P8 EPCs and P4 EPCs were defined as the senescent and young cell groups, respectively.

### Delivery of luciferase and VEGF pDNAs into EPCs by UMT

To evaluate the effect of UMT on young and senescent porcine EPCs in the presence of 3% (v/v) microbubbles, among the US parameters (intensity, 0.5–1 W/cm^2^; DC, 5–20%; exposure time, 30 s) used to perform UMT of luciferase pLuc-N3 pDNA and VEGF165 pDNA into endothelial lineage cells^29,34,36^, the optimal US parameters (intensity, 0.5 W/cm^2^; DC, 20%; exposure time, 30 s) were finally used for gene delivery into EPCs. UMT was performed 24 h after equal numbers of young and senescent EPCs were seeded into the experimental wells with 1000 μl of pDNA-containing growth medium per well. Subsequently, 48 h after UMT, a protein activity assay, ELISA, and cell counting for normalization of protein expression were performed.

**Figure 2:**
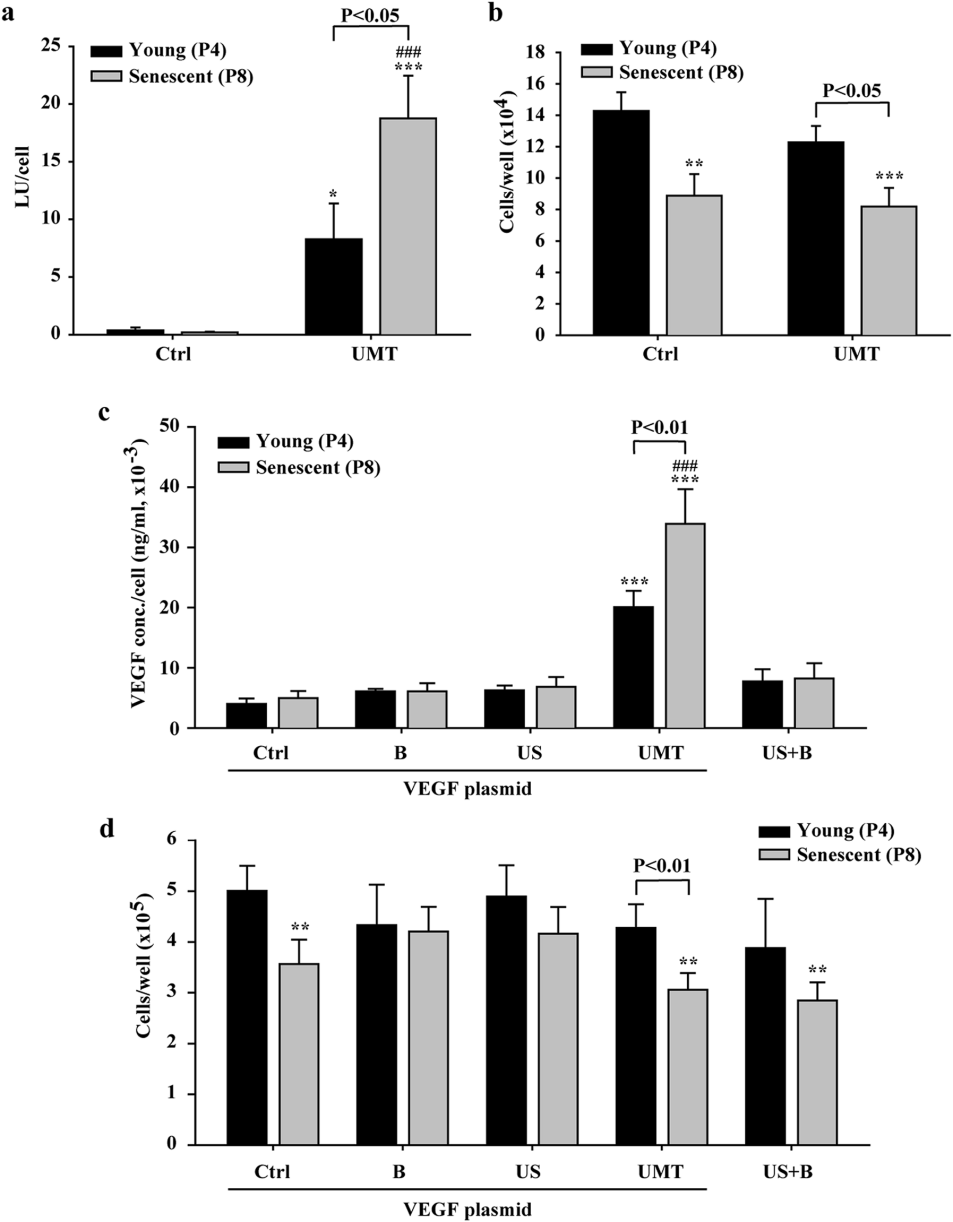
Efficacy of ultrasonic microbubble transfection (UMT, i.e., US + B + plasmid) in young and senescent EPCs and the effect on the cell number at 48 h after treatment, as evaluated by a luciferase assay (**a**), by cell counting with a Coulter counter (**b** and **d**) and by ELISA for VEGF expression (**c**). In (**c**) and (**d**), the cells were divided into 5 groups to evaluate the treatment effects of US and microbubbles in the presence or absence of plasmid on VEGF expression and the cell number. The VEGF plasmid was used in all groups except the “US + B” group. The luminescence intensity generated by luciferase expression and the expression level of the VEGF protein (in 1000 μl of pDNA-containing growth medium per well) are shown per cell (**a** and **c**). n = 9 for each bar. Note that (**b**) shows the number of remaining cells per well after UMT under the conditions used in (**a**), and that (**d**) shows the number of remaining cells per well after treatment under the conditions used in (**c**). Additionally, note that for the UMT groups, differences between the black and grey bars are indicated. *P < 0.05; **P < 0.01; ***P < 0.001 vs. the leftmost black bar and ^###^*P* < 0.001 vs. the leftmost grey bar. Ctrl = control. B = microbubbles. US = ultrasound. LU = luminescence unit.

The results showed that compared to the transfection without UMT, UMT enhanced luminescence generation, indicative of increased luciferase protein expression attributed to delivery of a greater amount of pDNA into EPCs, in both the young and senescent groups (both *P* < 0.05, Fig. 2a). In addition, there was a significant increase in luminescence generation in the senescent group with UMT compared to the young group with UMT (*P* < 0.05, Fig. 2a). The numbers of cells post treatment were similar between the young groups with and without UMT and between the senescent groups with and without UMT (*P* > 0.05, Fig. 2b). However, regardless of whether UMT was used, the cell number in the senescent group was lower than that in the young group (all *P* < 0.05, Fig. 2b), apparently due to ageing and the resulting weakening of the proliferative activity of senescent EPCs (Fig. 2b).

For VEGF transfection, cells were divided into 5 groups: a control group with pDNA (“Ctrl” group), a microbubble-only group (“B” group; with pDNA but no US), an US-only group (“US” group; with pDNA but no microbubbles), a UMT group (“UMT” group; with pDNA, US and microbubbles), and a US plus microbubbles group (“US + B” group; with US and microbubbles but without pDNA) (Fig. 2c and d). The US + B group was included to evaluate the mechanical effect of US plus microbubbles per se on endogenous VEGF synthesis in EPCs.

Similarly, a significant increase in VEGF expression was found in both the young and senescent groups subjected to UMT compared to the corresponding control groups (all *P* < 0.05, Fig. 2c). In addition, VEGF expression in the UMT group of senescent cells was significantly enhanced compared to that in the young control group (*P* < 0.001, Fig. 2c). In the UMT group, VEGF expression was significantly higher in the senescent cells than in the young cells (*P* < 0.01, Fig. 2c). The numbers of cells post treatment were similar among the young groups and among the senescent groups (*P* > 0.05, Fig. 2c). However, in the groups subjected to the same treatment, the cell number in the senescent subgroup was lower than that in the young subgroup (Fig. 2d), apparently due to ageing (Fig. 2d).

### Time-dependent changes in intracellular pDNA copy number and protein expression levels in EPCs post UMT

**Figure 3:**
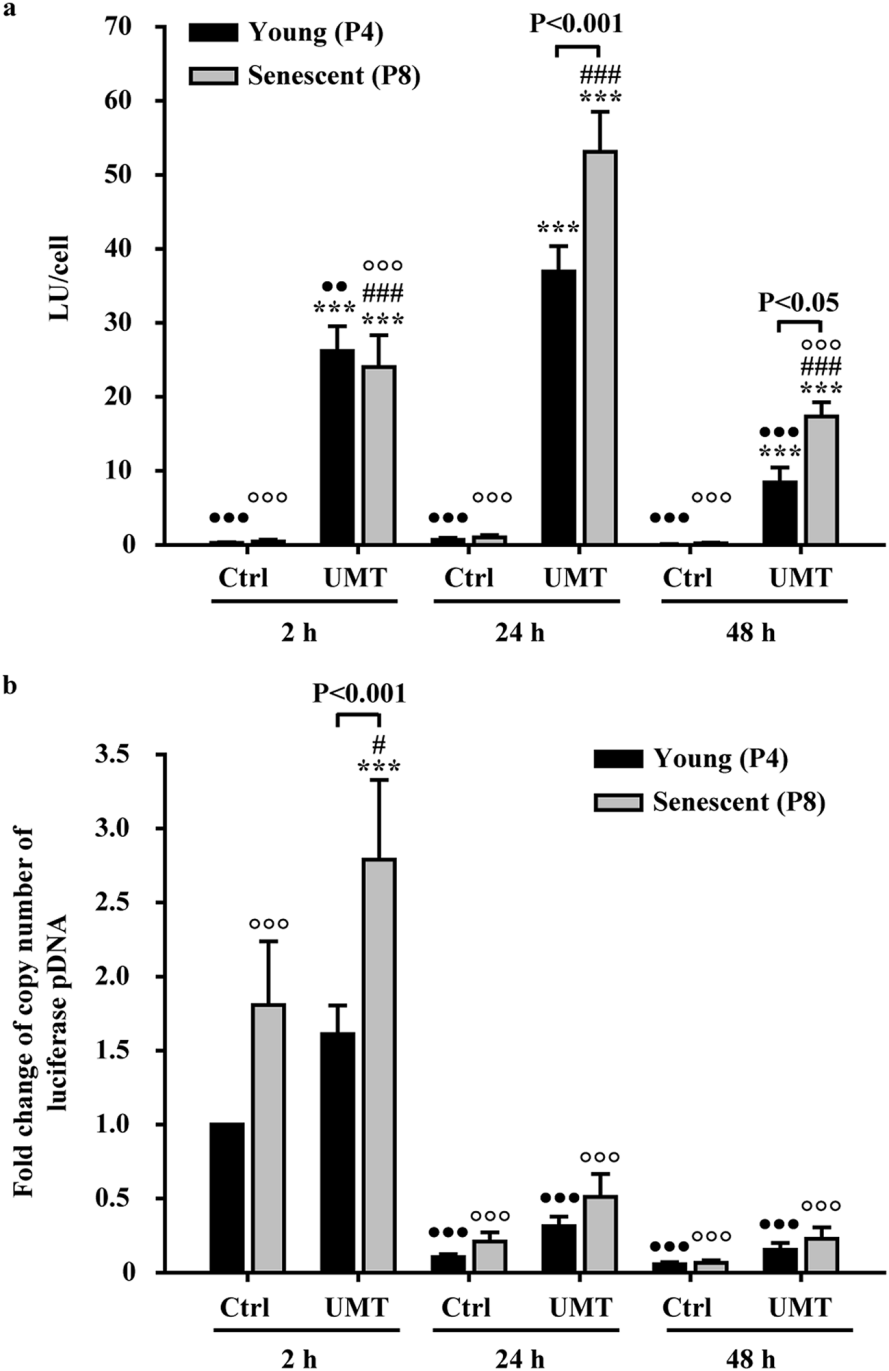
Comparison of the intracellular luciferase pDNA copy number and luminescence intensity in young and senescent EPCs at 2 h (h), 24 h and 48 h post UMT of luciferase pDNA. (**a**) The luminescence intensity, indicative of the luciferase protein level, was markedly increased in the UMT groups compared to the corresponding Ctrl groups at the same time points. In addition, in the UMT groups at 24 and 48 h, the senescent cells generated more luminescence than the young cells at the same time points. (**b**) Quantitative PCR analysis showed a higher luciferase pDNA copy number in the UMT groups than in the corresponding Ctrl groups at the same time points. In addition, the copy number was higher in senescent cells than in young cells at as early as 2 h post UMT. Thereafter, the copy number decreased markedly. See the text for details. n = 7 for each bar. ***P < 0.001 vs. the leftmost black bar at the same time point. ^#^*P* < 0.05; ^###^*P* < 0.001 vs. the leftmost grey bar at the same time point. ^••^*P* < 0.01; ^•••^*P* < 0.001 vs. the highest corresponding black bar and °°°*P* < 0.001 vs. the highest corresponding grey bar in (**a**) and (**b**). Note that for the UMT groups at the same time point, differences between the black and grey bars are indicated. All abbreviations are the same as those used in Fig. 2.

To clarify the cause of the changes in expression in EPCs post UMT, the intracellular pDNA copy number and protein levels or the protein activity of EPCs were sequentially evaluated in EPCs at the 2-h, 24-h and 48-h time points post UMT using the same protocol mentioned above. The results of luciferase reporter transfection of EPCs showed that, in the young cells, the luminescence intensity, reflecting the luciferase protein expression level, was significantly increased at all the 3 time points post UMT compared to that in the corresponding young control cells without UMT at the same time points (all *P* < 0.001, Fig. 3a). Consistent with this result, the luminescence intensity in senescent EPCs was also significantly enhanced at all the 3 time points post UMT compared to that in the corresponding senescent control cells without UMT at the same time points (all *P* < 0.001, Fig. 3a). Additionally, in the comparison between young and senescent cells at both the 24-h and 48-h time points post UMT, a higher luminescence intensity was noted in senescent EPCs than in the corresponding young EPCs (all *P* < 0.05, Fig. 3a). The strongest luminescence generated occurred at 24 h post UMT in both young and senescent EPCs (all *P* < 0.01, Fig. 3a).

The Q-PCR results showed that at each of the 3 time points, each individual UMT group had a higher intracellular pDNA copy number than the corresponding control group without UMT (*P* < 0.05 for the senescent group at the 2-h time point). In addition, at the 2-h time point post transfection, there were increased intracellular pDNA copy numbers in senescent cells compared to the corresponding young cells, with or without UMT (*P* < 0.001 for the UMT group, Fig. 3b). Furthermore, with or without UMT, the intracellular pDNA copy numbers were markedly decreased (more than 50%) in both young and senescent EPCs at the 24-h and 48-h time points post transfection compared to the corresponding copy numbers at the 2-h time point (for comparison with the highest values in the UMT groups, all *P* < 0.001, Fig. 3b).

**Figure 4:**
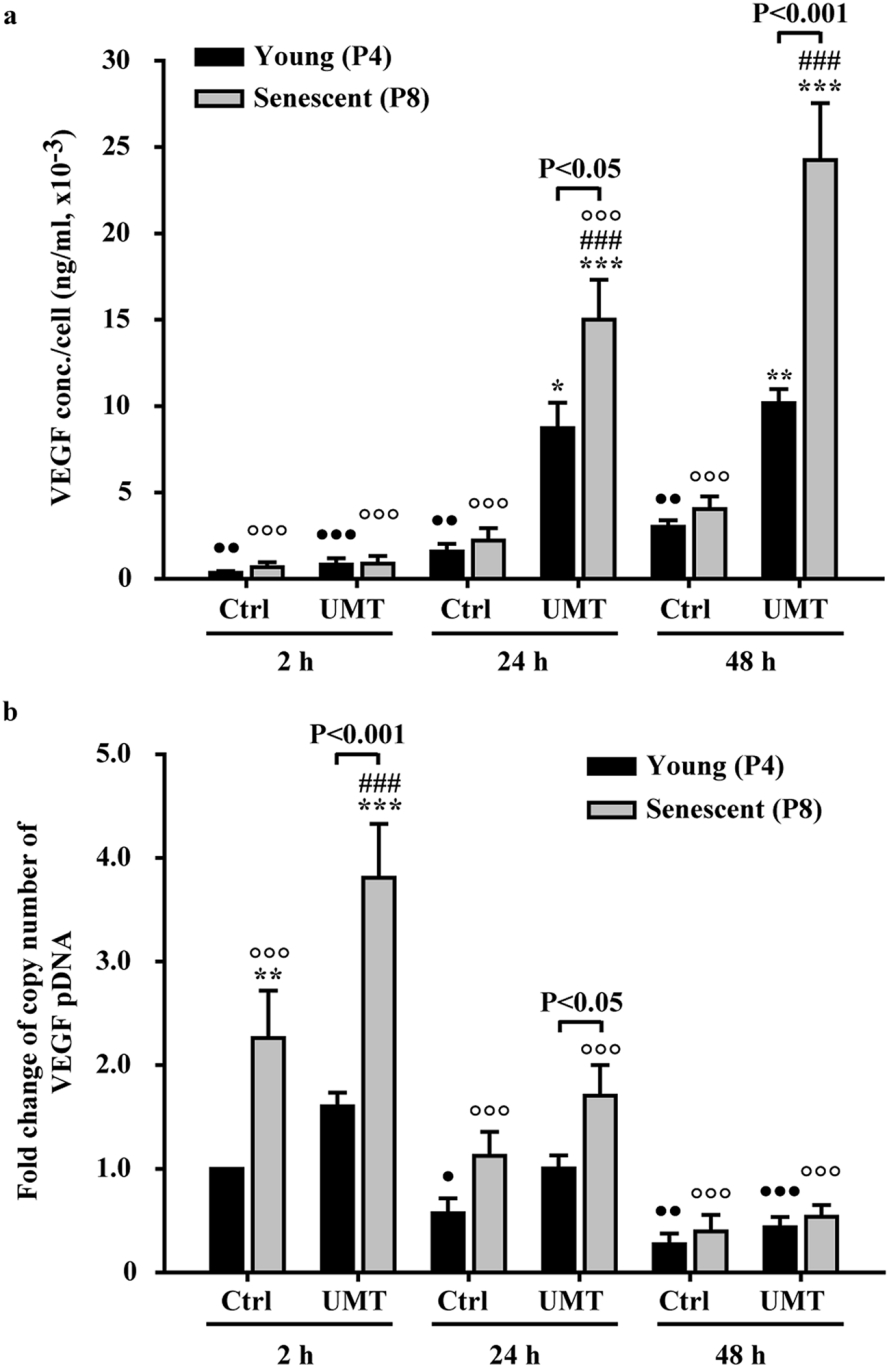
Comparison of VEGF protein levels in the supernatants (**a**) and intracellular pDNA copy numbers (**b**) at 2 h (h), 24 h and 48 h post UMT of VEGF pDNA into young and senescent EPCs. (**a**) ELISA and (**b**) quantitative PCR. See the text for details. n = 7 for each bar. *P < 0.05; **P < 0.01; ***P < 0.001 vs. the leftmost black bar at the same time point. ^###^*P* < 0.001 vs. the leftmost grey bar at the same time point. ^•^*P* < 0.05; ^••^*P* < 0.01; ^•••^*P* < 0.001 vs. the highest corresponding black bar and °°°*P* < 0.001 vs. the highest corresponding grey bar in (**a**) and (**b**). Note that for the UMT groups at the same time points, differences between the black and grey bars are indicated. All abbreviations are the same as those used in Fig. 2.

In EPCs with functional gene transfection of VEGF165, comparison (Fig. 4) was performed between the control and UMT groups at different time points, since the significant effect of VEGF pDNA transfection indicated in Fig. 2c was only noted in the UMT groups of young and senescent cells. The results showed that the variations in both VEGF protein expression (Fig. 4a) and the intracellular pDNA copy number (Fig. 4b) also resembled the patterns seen with luciferase transfection except that in the transfected cells, the increased in protein expression occurred more slowly and peaked at 48-h post UMT (Fig. 4a).

### Angiogenic properties of EPCs post US exposure

**Figure 5:**
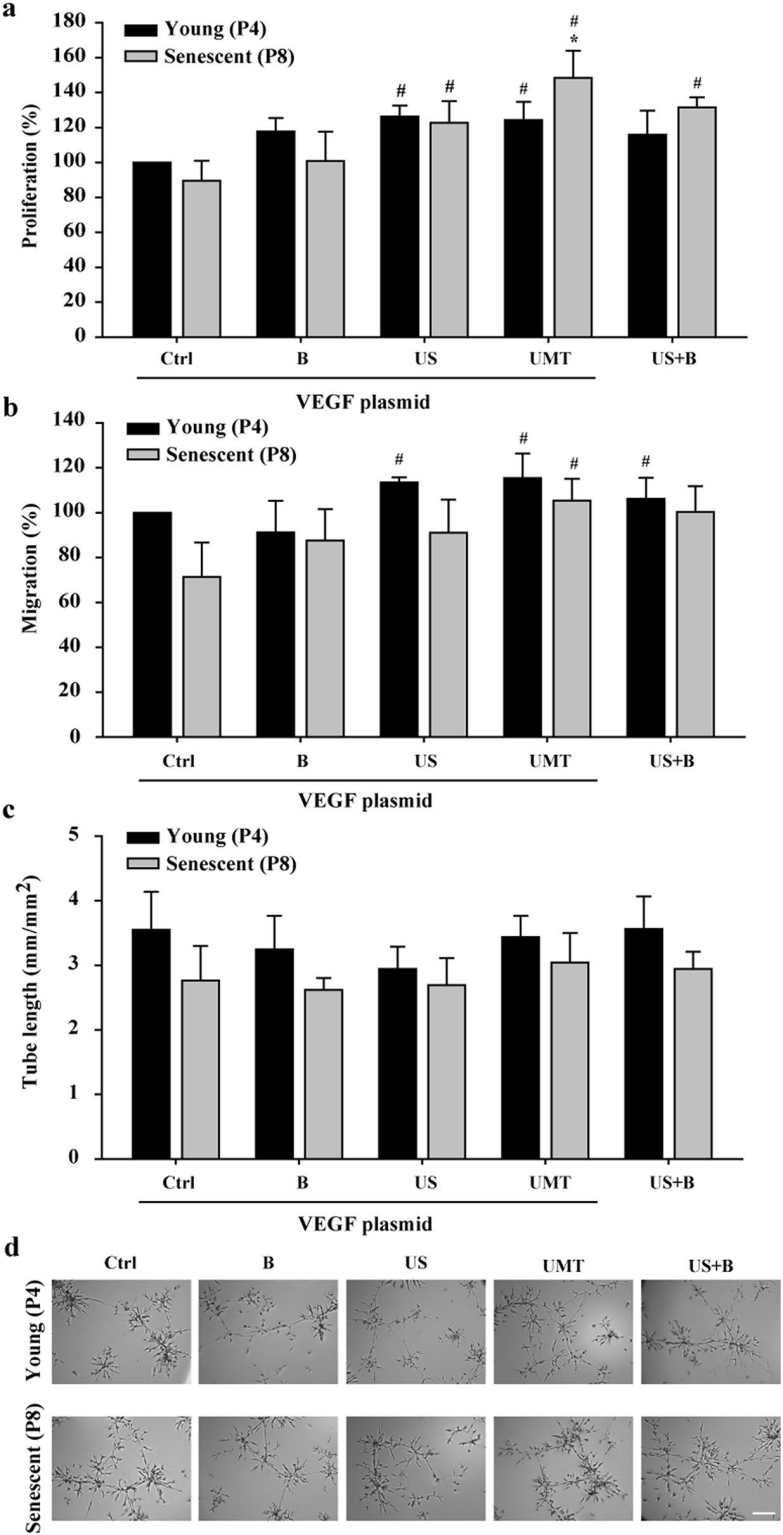
Effects of UMT-mediated VEGF transfection on the angiogenic activities of transfected young and senescent porcine EPCs. At 48 h after transfection, the cells were evaluated for proliferation by a BrdU incorporation assay (**a**), by a migration assay (**b**), and by a tube formation assay (**c**). Representative micrographs are shown (**d**). Scale bar: 250 µm. Migration and tube formation were analysed with Leica QWin image analysis software (Cambridge, UK, version number: V3.5.2). See the text for details. n = 6 for each bar. *P < 0.05 vs. the leftmost black bar. ^#^*P* < 0.05 vs. the leftmost grey bar. All abbreviations are the same as those used in Figs. 1 and 2.

To investigate whether UMT-mediated VEGF transfection alters the angiogenic activity of the transfected EPCs, the proliferation, migration, and tube formation activities of EPCs were evaluated 48 h post VEGF transfection (Fig. 5). From the results of previous optimization experiments, regardless of whether only pDNA was added, there were no changes in the angiogenic activity between young and senescent EPCs (see supplementary material, Fig. 3). To analyse the effects of US and microbubbles alone or in combination on EPCs, groups similar to those shown in Fig. 2c,d were established with the cells used for the assays shown in Fig. 5: a control group with pDNA (“Ctrl” group), a microbubble group with pDNA (“B” group), a US group with pDNA (“US” group), a UMT group with plasmid DNA (“UMT” group), and a US plus microbubbles group without pDNA (“US + B” group; see Fig. 5).

The results showed that in the senescent cells, compared to the group with pDNA only (“Ctrl”), the B group, US group, UMT group and US + B group showed a trend of enhanced or significantly enhanced migration and proliferation activities (Fig. 5a-b). Regarding the comparison between young and senescent cells, the proliferation and migration abilities of young cells showed an increasing trend or a significant increase compared to those of the senescent control cells (Fig. 5a,b). Regarding tube formation activity, there was an increasing trend in all young cell groups in comparison to their corresponding senescent groups, but the difference were not significant (*P* > 0.05, Fig. 5c). Surprisingly, proliferation activity in the UMT group of senescent cells was significantly higher than that in the young control group (*P* < 0.05, Fig. 5a).

### Biological effects of the supernatant from UMT-mediated VEGF-transfected EPCs on HAECs

**Figure 6:**
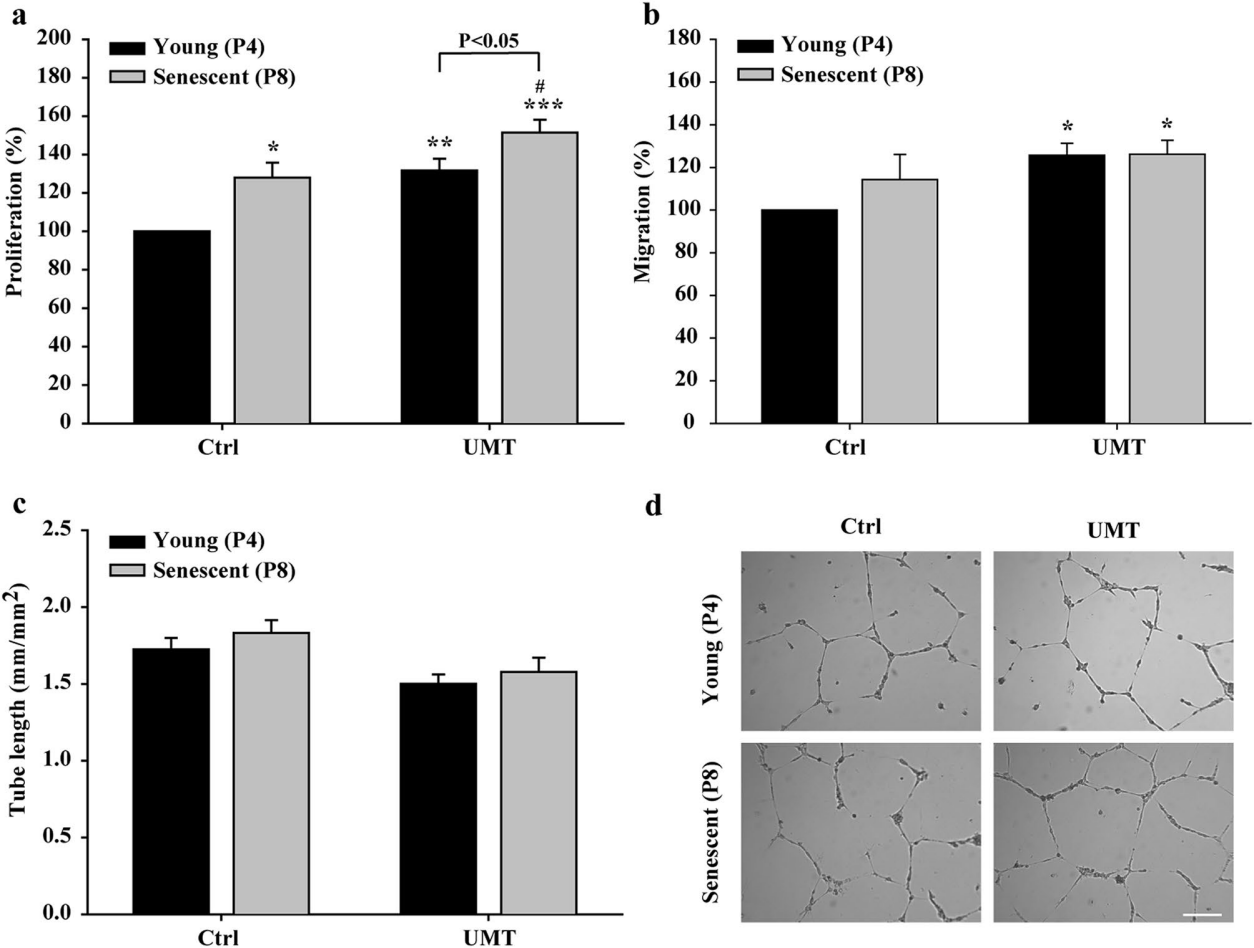
Effects of supernatants (1000 μl/well) from young and senescent porcine EPCs 48 h post UMT-mediated VEGF transfection on the proliferation (**a**), migration (**b**) and tube formation (**c**) of human aortic endothelial cells. (**d**) Representative micrographs of tube formation in the different groups are shown. Scale bar: 250 µm. Proliferation was evaluated using a BrdU incorporation assay. Migration and tube formation were analysed with Leica QWin image analysis software (Cambridge, UK, version number: V3.5.2). n = 7 for each bar. In **a**, **b** and **c**: *P < 0.05; **P < 0.01; ***P < 0.001 vs. the leftmost black bar and ^#^*P* < 0.05 vs. the leftmost grey bar. Note that for the UMT groups, differences between the grey and black bars are indicated. All abbreviations are the same as those used in Fig. 2.

The biological effects of VEGF-containing supernatant from transfected EPCs (as measured in Fig. 4a) on the angiogenic activities (proliferation, migration and tube formation) of HAECs were evaluated in vitro (Fig. 6). Since the significant effect of VEGF pDNA transfection indicated in Fig. 2c was only noted in the UMT groups of young and senescent cells, only the control and UMT groups were compared (Fig. 6). The results showed that for supernatants from cells without UMT, the proliferation activity of HAECs was significantly enhanced by supernatants from senescent EPCs compared to those from young cells (*P* < 0.05, Fig. 6a). In addition, compared to supernatants from the corresponding EPCs without UMT, supernatants from young and senescent EPCs with UMT enhanced the proliferation of HAECs (*P* < 0.05, Fig. 6a). Furthermore, for supernatants from cells with UMT, enhanced proliferation activity of HAECs was also seen for supernatants from senescent EPCs compared to supernatants from young EPCs (*P* < 0.05, Fig. 6a). The migration activity of HAECs was significantly enhanced by supernatants from both young and senescent EPCs with UMT compared to supernatants from the young control EPCs without UMT (*P* < 0.05, Fig. 6b). However, the tube formation activity did not differ between groups (*P* > 0.05, Fig. 6c).

## Discussion

In this study, a serial passage model of senescence was established to mimic the ageing process of porcine EPCs, and 4 questions were answered: (1) Can the gene encoding the growth factor VEGF be successfully delivered into senescent EPCs by UMT? (2) Is the effect of VEGF gene delivery different between young and senescent EPCs? (3) What are the time-dependent changes in the intracellular pDNA copy number and protein expression levels in EPCs post UMT? (4) What are the phenotypic alterations in EPCs and the angiogenic effects of the supernatants obtained from cells post UMT-mediated functional gene delivery, and what are the clinical implications of these findings? The key results demonstrated that UMT-based VEGF gene delivery can augment the angiogenic effect of EPCs, even in senescent cells, and may provide a foundation for enhancing the beneficial effects of cell therapy when only senescent EPCs are available.

In the present study, senescent EPCs were defined as EPCs with replication-induced senescence at a modest level. Compared with P4 EPCs, P8 EPCs were found to have an increase in the cell doubling time of more than 50% with significant shortening of telomere length^37^, accompanied by an increase of up to 400% in acidic *β*-galactosidase activity^38,39^, and consequently, the P8 and P4 EPCs were defined as senescent and young cells, respectively. Cell therapy requires an adequate number of cells with proper cellular function. When cells were propagated from P4 to P8 in the present study, the cell number was increased more than 50-fold. The aim of this study was to improve the angiogenic effect of senescent EPCs by delivery of the UMT-based growth factor gene VEGF165.

Our previous report showed that the VEGF gene can be delivered into young EPCs by UMT^36^. The present study further showed that the effect of transfection existed only in the UMT group and that without the plasmid, ultrasonic microbubble delivery alone did not cause a significant increase in the secretion of endogenous VEGF from EPCs. By UMT with a list of optimized US parameters, both the luciferase reporter and functional VEGF165 pDNA were successfully delivered into EPCs, even senescent EPCs.

To our knowledge, this is the first report showing that senescent EPCs express higher levels of a pDNA-derived protein than young cells after the same ultrasonic microbubble delivery procedure. The decreased numbers of remaining cells after transfection seen in the present study were attributed to senescence of EPCs.

In efforts to understand the time-dependent changes in the intracellular pDNA copy number and protein expression levels in EPCs post UMT, sequential pDNA transfection was performed after determination of a set of optimized US parameters that are necessary to achieve a balance between the efficiency of gene delivery and UMT-associated mechanical damage to EPCs. In our present report, the significant enhancement of both luciferase and VEGF protein expressions was noted at different time points in the UMT groups in comparison to the corresponding control with pDNA groups. There are multiple cellular barriers impeding the nuclear accumulation of pDNA or DNA-vector complexes that need to be overcome during non-viral gene transfer, including cell binding, cell entry/endocytosis, endosome escape, cytoplasmic transport, and nuclear entry^40^. Although nuclear transport of the therapeutic DNA is known to be the critical determinant of transgene expression during the gene delivery process^41^, many researchers have challenged the view of a single ‘rate-limiting’ barrier to efficient transgene expression^42,43^. Fasbender A et al*.* showed that reduced DNA uptake limited cationic lipid-mediated gene transfer in primary cultures of human airway epithelial cells^44^. A study by Lehmann MJ et al*.* showed that the apparent increase in the cellular uptake of biologically active recombinant DNA was accompanied by an apparent increase in the expression of luciferase^45^. In addition, Kong Q et al. reported a significant correlation between the copy number of pDNA and the transgene expression level in vitro and in vivo^46^. The results of our present study demonstrated that a significant increase in the intracellular reporter or functional pDNA copy number at the early 2-h time point after UMT-based gene delivery could be an important determinant of transgene expression in EPCs. Importantly, in the UMT groups at the early 2-h time point, the significant increase in the pDNA copy number in the senescent group was expected to cause enhanced protein expression in senescent cells in comparison to young cells post UMT. There is no literature discussing this contrasting effect in young and senescent EPCs post UMT, but one particularly influential proposal (the membrane hypothesis of ageing) suggests a cellular mechanism based on the observation that more compact cellular structures, such as cell membranes, are compromised faster than more distributed ones, such as the cytosol, during the ageing process^47^. Biological membranes in eukaryotic cells are dynamic structures that generally consist of bilayers of amphipathic molecules held together by noncovalent bonds^48^. In our study, ultrasonic microbubble-mediated gene delivery induced transient pore formation in cell membranes (i.e., sonoporation)^21,26–28^ and allowed the entry of reporter pDNA and VEGF pDNA into the cells. Therefore, according to the membrane hypothesis of ageing, the biological effect of inertial cavitation induced by UMT-based delivery may enhance pore formation on the membrane of senescent EPCs in comparison to the membrane of young cells and subsequently cause an increase in the pDNA copy number in senescent EPCs post UMT, especially at the early 2-h time point. Therefore, a significant difference in protein expression between the young and senescent groups at different time points post UMT existed.

Regarding the intracellular pDNA copy number post transfection, in contrast to virus-induced stable transfection where transgenes are integrated into the host genome and their expression is maintained even after the host cell replicates^49,50^, UMT induces a transient gene expression state in which pDNA does not integrate into the nuclear genome and is not passed on to daughter cells^40,51^. As a result, introduced genetic materials, such as intracellular pDNA, are diluted through mitosis or degraded^52^. Gene expression changes can be generally studied in a limited window of 8 to 96 h post transfection^29,53,54^. In our study, in the UMT groups, a significant increase in protein expression was noted between 24 and 48 h in the VEGF group but was noted earlier in the luciferase group than in the corresponding control with pDNA group. Transfection experiments using luciferase as the reporter protein are an essential method to examine the initial effect of gene delivery tools, including UMT. On the other hand, one may question why the peak expression of luciferase and VEGF occurred at different time points in the present study. Detection of luciferase is rapid and highly sensitive, because it can be detected intracellularly (i.e., it does not need to be secreted into the extracellular space before detection). Unlike luciferase, the VEGF detected here included the protein synthesized by the transfected VEGF165 and the protein synthesized by endogenous DNA. We did not know the exact length of time required for the synthesis of both proteins. However, since VEGF was detected by ELISA, the protein must be secreted into the extracellular space, and the time needed for detection of VEGF is thus longer than that required for detection of luciferase. A higher intracellular pDNA copy number was noted in the senescent control group than in the young control group at the 2-h time point post transfection. According to the membrane hypothesis of ageing, cellular ageing affects the plasma membrane^47^, which may explain why the membrane of senescent cells allows pDNA to pass through more readily than the membrane of young cells. Future electron microscopy studies will be required to clarify this issue.

Since late EPCs augment angiogenesis by directly incorporating into developing vascular networks and exhibiting paracrine effects, the influence of UMT on the abovementioned two dimensions of angiogenic activities was assessed in this study. In our previous report, there was little change in the angiogenic properties of young EPCs post UMT of the VEGF plasmid^36^. In contrast, in the present study, comprehensive testing by various protocols and additional experiments indicated that with the optimized US parameters, both young and senescent EPCs exhibited enhanced migration and proliferation activities post UMT of the VEGF plasmid. Additionally, the mechanical effects of US plus microbubbles per se without the addition of pDNA improved the proliferation activity of senescent EPCs. In the literature, after DNA (hepatocyte growth factor, HGF)/ultrasound/microbubble therapy, an increased angiogenic effect was observed in a rabbit hindlimb ischaemia model^27^. Cardiac US-mediated HGF delivery in a rat model of myocardial infarction improved cardiac angiogenesis and left ventricle function^55^. Moreover, enhancement of capillary perfusion following low-intensity US with microbubbles but without pDNA was noted during postischaemic reperfusion in hamsters^56^. For the first time, our results showed that US plus microbubbles with or without VEGF can improve the angiogenic properties, including the proliferation, and/or migration activities, of senescent EPCs.

In the present study, the reason for the improved angiogenic activities of HAECs treated with the supernatant of senescent EPCs post UMT is not well understood. However, the increase of up to 100% in VEGF production per cell in the supernatant of senescent cells post UMT observed in the present study provides insight. VEGF is primarily reported to play a central role in the regulation of angiogenesis and exerts protective effects against cell damage, including damage from senescence and senescence-associated ischaemic hypoxia^57–61^. Therefore, the enhanced effect of the supernatant from senescent cells on HAEC function was presumed to be mainly due to ageing of the plasma membrane of EPCs^47^, which allows a greater amount of VEGF pDNA to pass through for transgene expression, especially post UMT. The findings that the angiogenic activities of senescent EPCs improved after UMT suggests that a future in vivo experiment, e.g., a mouse model of hindlimb ischaemia with EPC therapy in the ischaemic limb, is worthwhile to test the potential for clinical application and to verify the enhanced therapeutic effects of senescent EPCs post UMT compared to young and senescent cells with or without UMT.

## Conclusions

The present report illustrates the potential of UMT-mediated delivery of growth factors, such as VEGF, as a non-invasive method to augment the angiogenic effect of senescent EPCs. Such a strategy may compensate for the functional impairment of cells after ex vivo expansion to obtain an adequate number of cells. Further investigations are required to determine the enhancing effect of senescent EPCs on angiogenesis in vivo.

## Methods

### Cell culture

The design of all methods in this study was in accordance with the guidelines in the Declaration of Helsinki. Porcine EPCs were propagated and used in the present experiments. Two cell types, HAECs (Invitrogen, Carlsbad, CA, USA) and EPCs, were propagated and used in the experiments.

Isolated EPCs were collected from peripheral blood samples of Taiwanese Lanyu miniature pigs (*Sus barbatus sumatranus*) (15–26 kg, male)^36^. This design of the collection protocol was approved by the Institutional Animal Care and Use Committee (IACUC, Approval No: MMH-A-S-102-66), which is the ethics committee/institutional review board of MacKay Memorial Hospital, Taipei, Taiwan. The study was carried out in compliance with the ARRIVE guidelines. Fifty millilitres of venous blood was centrifuged at 400 × g for 15 min. After collection of the buffy coat, peripheral blood mononuclear cells (PBMCs) were isolated by Ficoll-Paque PLUS (GE Healthcare, Uppsala, Sweden) according to the manufacturer’s instructions. The cells were then washed with 0.9% normal saline and centrifuged at 200–400 × g for 5 min. Finally, PBMCs were resuspended in MV2 endothelial cell growth medium (PromoCell, Heidelberg, Germany) with SupplementMix (PromoCell) and cultured on 6-well plates (Corning, NY, USA) coated with 1 μg/cm^2^ human fibronectin (Corning, Bedford, MA, USA) in a 5% CO_2_ incubator at 37 °C. Cobblestone-like cell colonies were observed after porcine PBMCs were cultured in MV2 medium for 10–14 days. The colonies were trypsinized with 0.05% trypsin-ethylenediaminetetraacetic acid (Gibco, Grand Island, NY, USA) for passage. At 70 to 80% confluency, EPCs were subcultured at a dilution ratio of 1 to 3. Young passage (P) 4 EPCs (1.5 × 10^6^ cells/culture dish) and senescent P8 EPCs (1 × 10^6^ cells/culture dish) were used for individual experiments after reaching 90% confluency.

### Immunofluorescence for characterization of EPCs

EPCs were seeded at a density of 3 × 10^4^ cells on cover glasses (12 mm diameters, Deckglaser, Sondheim, Germany). Cells at 80% confluence were fixed with 4% paraformaldehyde (EM grade, Electron Microscopy Sciences, PA, USA) for 10 min, followed by 3 PBS washes. Cells were blocked with 10% horse sera for 1 h and incubated with primary antibodies (diluted at 1:100 × with PBS-TX100 (0.2%)) at 4 °C overnight. After 3 washes with PBS-TX100 (0.2%), the cells were incubated with the corresponding secondary antibodies at room temperature for 3 h. Cell nuclei were stained with DAPI (4´,6-diamidino-2´-phenylindole (1 mg/ml), 3000X diluted) for 10 min, followed by 3 washes with PBS-TX100. The cover glasses with cell cultures were removed from 24-well plates and mounted for imaging acquired by a Leica TCS SP8 confocal microscope (Leica Microsystems, Wetzlar, Germany). Antibodies against PECAM1 (CD31, #MAB1398Z) were purchased from EMD Millipore (CA, USA). Antibodies against VE-cadherin (sc-9989) were purchased from Santa Cruz (TX, USA). Antibodies against vWF (F8/86) were purchased from Dako (Denmark). Antibodies against eNOS (D9A5L) were purchased from Cell Signaling Technology (MA, USA). Fluorescein isothiocyanate (FITC)-labelled *Ulex europaeus agglutinin*-1 (UEA-1) lectin (GTX01512) was purchased from GeneTex (CA, USA). DiI-acetylated low density lipoprotein (DiI-ac-LDL) (L3484) was purchased from Thermo Fisher (MA, USA). Cells were incubated with DiI-ac-LDL at 37 °C for 4 h and fixed with 4% paraformaldehyde for 10 min. After two washes, the cells were stained with FITC-labelled UEA-1 at room temperature for 2 h.

### Plasmid DNA preparation

This study used 2 pDNA vectors. The luciferase pLuc-N3 (cytomegalovirus (CMV) promoter, 5668 base pairs) derived from pEGFP-N3 (Clontech Laboratories, Mountain View, CA, USA; green fluorescent protein (GFP)) and the pGL3-Control Vector (Promega, Madison, WI) were used to evaluate reporter gene expression^36^. The VEGF165 plasmid was used for functional gene transfection. A solution of pDNA diluted to a concentration of 15 mg/mL was used in the transfection medium. The luciferase pLuc-N3 vector was purified with an endotoxin-free plasmid DNA Kit (Machery-Nagel, Düren, Germany) according to the manufacturer’s instructions. For construction of the pShuttle-CMV/VEGF plasmid, a human VEGF165 cDNA fragment was digested from the phVEGF165 plasmid^36^. The full-length cDNA fragment was cloned into a pCA3 vector (Microbix Biosystems, Toronto, ON, Canada), followed by HindIII and XbaI double digestion and subsequently cloned into a pShuttle-CMV vector (Agilent Technologies, Santa Clara, CA, USA). VEGF expression was ultimately verified by sequencing.

### Estimation of EPC doubling time

EPC doubling time was determined by Cell Counting Kit-8 (CCK-8; Invitrogen, Carlsbad, CA) assays based on the conversion of a highly water-soluble tetrazolium salt, WST-8, [2-(2-methoxy-4-nitrophenyl)-3-(4-nitrophenyl)-5-(2,4-disulfophenyl)-2H-tetrazolium, monosodium salt], to a water-soluble formazan dye upon reduction by dehydrogenases in the presence of an electron carrier^62^. EPCs (2 × 10^4^ cells/ml) were grown on 24-well plates. Fifty microlitres of CCK-8 solution was added to each well at the 0, 22nd, 46th, and 70th hours, followed by a 2-h incubation at 37 °C. The absorbance at 450 nm was measured by a multiplate reader (Lambda Bio-20; Beckman). This analysis was performed every day, with four technical replicates conducted each time during the exponential phase.

### Senescence-associated *β*-galactosidase activity assay

Senescent cells were measured by using a cellular senescence assay kit (SA-βgal staining; cat. no. CBA-231 Cell Biolabs)^63^. Briefly, cells were washed twice with 3 ml of PBS, resuspended in cell lysis buffer, and then transferred to a 96-well plate. Equal volumes of freshly prepared 2 × assay buffer were added to each well and incubated at room temperature for 2 h in the dark. After staining, fluorescence was measured by a fluorescence plate reader at 360 nm (excitation)/465 nm (emission).

### Telomere length assay

Genomic DNA was extracted from porcine late EPCs using a high purity PCR template preparation kit protocol (Roche, USA)^64^. The average telomere length was measured from total genomic DNA using quantitative real-time polymerase chain reaction (Q-PCR) with murine telomere primers: (forward), CGGTTTGTTTGGGTTTGGGTTTGGGTTTGGGTTTGGGTT and (reverse), GGCTTGCCTTACCCTTACC CTTACCCTTACCCTTACCCT. Primers for the reference control gene (porcine 36B4 single-copy gene) were as follows: (forward), TGAAGTGCTTGACATCACCGAGGA and (reverse), CTGCAGACATACGCTGGCAACATT. The PCR amplification system for telomeres included a SYBR Green Mix of 10 μl and 400 nM forward and reverse telomere primers. The reaction system for the 36B4 gene contained the same amount of SYBR Green mix as telomeres, 400 nM forward primer and 640 nM reverse primer. Samples with equal amounts (20 ng) of DNA were added to 2 adjacent wells. A sufficient quantity of double-distilled water was added to each well to yield a final volume of 20 μl. Both telomeres and the 36B4 gene were amplified under the same conditions. All PCRs were performed using an ABI StepOne Real-Time PCR System (Applied Biosystems; Thermo Fisher Scientific, Inc.). The telomere (T) signal was normalized to the signal from the single-copy (S) gene to generate a T/S ratio indicative of relative telomere length^64^. Equal amounts of DNA (20 ng) were used for each reaction, with at least 3 replicates for each specimen.

### Microbubble preparation and ultrasound transfection

Hard-shell microbubbles (BG8187) were made of tripalmitine (a triacylglyceride) and gassed with air (1.5–2.5 μm in diameter; Bracco, Geneva, Switzerland)^29^. Microbubbles were added to the transfection medium (500 μl/well) at a concentration of 3% (volume (v)/v), yielding a bubble number of approximately 4.66 × 10^7^ bubbles/well in 500 μl of transfection medium.

Once the cell confluency reached 80%, EPCs were plated on fibronectin-coated 24- or 6-well plates (Corning) at a density of 3 × 10^4^ (or 1.5 × 10^5^) cells per well 24 h before transfection (day 1). The transfection procedures were performed on day 2. The total transfection duration was 2 h for each experiment on day 2. US-mediated transfection was performed 30 min after adding the transfection medium. Before US exposure, the cells were washed once with Hank's balanced salt solution without Ca^2+^ and Mg^2+^ (Gibco) and then incubated in the plasmid (15 μg/ml)-containing transfection medium at 37 °C for 30 min in a 5% CO_2_ humidified incubator. After the US exposure, the cells were allowed to rest for another 1.5 h, washed with FBS (5%)-containing MV2 medium three times to remove the undelivered plasmids, and then cultured in FBS (5%)-containing MV2 medium for 48 h for the following experiments^34,36^.

US-mediated transfection was performed using a Sonitron 2000 system (Rich-Mar, Inola, OK, USA)^29,34,36^. The US probe was slotted directly into the cell suspension in a culture well, with the transducer 2 mm below the transfection medium. The culture plate was suspended in a polystyrene water bath at 37 °C during transfection. The water bath (20 cm in depth) had a corrugated silicone mat fixed to the bottom of the water tank that allowed sufficient space for the reflected waves to escape without interfering with the rest of the plate. With a probe (10 mm in diameter), the length of the near field of the transducer was measured as approximately 16 mm. Immediately after the addition of microbubble-containing transfection medium into the well, the cells were sonicated by 1.0-MHz US with exposure times of 30 s at an intensity of 0.5–1 Watt (W)/cm^2^ and a duty cycle (DC) of 5–20%^29,34,36^. Approximately 20% of the microbubbles in the well were destroyed after US exposure (control vs. UMT, 100% ± 1.3% vs. 81.03% ± 0.62%, *P* < 0.01, see supplementary material, Fig. 1). The selected US parameters (0.5 W/cm^2^/DC 20%/exposure time 30 s) with a peak rarefactional pressure of 1 megapascal (MPa) and pulse repetition frequency of 250 Hz were finally used in the gene delivery of EPCs. See Supplementary Fig. 2 for the experimental setup of US exposure.

### Firefly luciferase activity assay and cell viability

The firefly luciferase activities of transfected EPCs were measured using a luciferase kit (Promega, Madison, WI, USA) and luminometer (Hidex Oy, Turku, Finland). For normalization of the luciferase activity to the number of remaining cells post-transfection in each well, a cell count was performed with a Coulter Counter (Beckman, Brea, CA, USA) 2 h, 24 h and 48 h after transfection. The final luciferase expression in each group is presented as light units/cell.

### Enzyme-linked immunosorbent assay

Enzyme-linked immunosorbent assays (ELISAs) were performed to quantify the VEGF protein in the medium after transfection^36^. The culture supernatants that were harvested from EPCs at different passages 2 h, 24 h and 48 h after UMT were collected and stored at − 80 °C. The amount of VEGF in each supernatant was quantified by a human VEGF immunoassay kit (R&D Systems, Minneapolis, MN, USA) according to the recommended procedures. The optical density was measured by a SpectraMAX 190 absorbance microplate reader (Molecular Devices, Sunnyvale, CA, USA) at 450–540 nm.

### Measurement of intracellular pDNA copy number

Total DNA, including genomic DNA and pDNA, was purified with a PCR purification kit (High Pure PCR template preparation kit, Roche)^62^. PCR amplifications were performed in a final reaction volume of 20 µl containing 10 µl of Universal PCR Master Mix (Roche, USA), 200 nM primers and 20 ng DNA. The primers were designed to target pDNA as follows: pLuc, (forward) TGATTGACAAGGATGGATGG and (reverse) GGTGGTGGAGCAAGATGG; phVEGF165, (forward) TGATGAGATCGAGTACATCTT and (reverse) TTCACATTTGTTGTGCTGTAG. Plasmid DNA per genome was then calculated according to the ratio of pDNA copy number to the measured single-copy gene 36B4^62^.

### Cell proliferation, migration and tube formation assays

Cell proliferation was determined by using a bromodeoxyuridine (BrdU) cell proliferation assay kit (Calbiochem, USA), which detected the presence of BrdU incorporated into cellular DNA during cell proliferation in experiments measuring the biological effect of VEGF secreted from transfected EPCs on HAEC proliferation^34,36^. For this purpose, HAECs, 10^4^ cells in 500 μl of supernatant from transfected EPCs at 48 h post-transfection, were seeded. Proliferation was measured by a SpectraMAX 190 absorbance microplate reader (Molecular Devices) at dual wavelengths of 450 to 540 nm.

The Boyden chamber assay was applied to evaluate cell migration in 24-well transwell chambers (8 μm pore size, Corning)^34,36^. The Transwell assay was based on a chamber of 2 medium-filled compartments separated by a microporous membrane. During the experiments to evaluate the effects of supernatants from the transfected EPCs on the migration of HAECs, HAECs (3 × 10^4^ cells in 100 μl of cell growth medium MV2) were seeded in the upper compartment, and 600 μl of supernatant (with 1% foetal bovine serum (FBS)) from the transfected EPCs at 48 h post-transfection was added to the lower compartment. In contrast, in the experiments to evaluate the migration of transfected EPCs (3 × 10^4^ cells in the upper compartment), 100 μl of FBS (0.5%)-containing MV2 was added to the upper compartment, and 600 μl of FBS (2.5%)-containing MV2 was added to the lower compartment. After incubation at 37 °C for 4 h, the membrane between the 2 compartments was fixed (− 20 °C methanol for 5 min) and stained with 18.7 mM bisbenzimide (Sigma, Missouri, USA for 20 min). Next, the number of cells that had migrated to the lower side of the membrane was determined by applying inverted fluorescence microscopy (Leica, Wetzlar, Germany) at × 50 magnification and analysed by Leica QWin image analysis software (Cambridge, UK, Version number: V3.5.2). Then, the cells were counted to determine the average number of cells that experienced transmigration.

Concerning the percentage that was calculated, the results of angiogenic activities (migration or proliferation) in the individual young control group in each histogram of Figs. 5a,b, 6a,b were regarded as 100%, and subsequently, the findings of the other study groups in the same histogram are represented as a percentage of those of the control group.

In terms of the tube formation assay, 200 μl of Matrigel (BD Biosciences, Bedford, MA, USA) was added on a 24-well plate for 30 min to allow the gel to solidify and thus prepare it for the tube formation assay in 2 experiments either concerning either UMT on the tube formation activity of transfected EPCs or measuring the biological effect of VEGF secreted from transfected EPCs on tube formation of HAECs^34,36^. For transfected EPCs, cells were resuspended in MV2 containing 2% FBS and were seeded at a density of 3 × 10^4^ cells per well onto Matrigel-coated wells. For HAECs, 3 × 10^4^ cells in 500 μl of supernatant from transfected EPCs at 48 h post-transfection were seeded onto Matrigel-coated wells. These cells were incubated at 37 °C for 24 h to form capillary-like structures. The cumulative tube length was calculated in 4 randomly selected microscopic fields at 50 × magnification derived from 4 independent experiments. Tube length was measured using Leica QWin image analysis software (Cambridge, UK, Version number: V3.5.2) as an indicator of angiogenic potential.

### Statistical analysis

All data are expressed as the mean ± SEM. Experimental groups were compared for statistical significance using a one-way analysis of variance (ANOVA), followed by Fisher's least significant difference test for multiple comparisons if the number of study groups was three or more. A *p* value of < 0.05 was considered to indicate statistical significance. The n number shown refers to the number of separate experiments; on each occasion, each treatment was performed at least in triplicate wells. All experiments were performed at least four times.

### Ethical approval

All authors have read and approved the submission of the revised manuscript; the revised manuscript has not been published and is not being considered for publication elsewhere, in whole or in part, in any language, except as an abstract.

## Supplementary Information

Supplementary Information 1.

Supplementary Information 2.

Supplementary Information 3.

Supplementary Information 4.

## Data Availability

All data supporting findings of this study are available within the article or from the corresponding author upon request.
