# Supplementary figures and images for: Ultrasonic microbubble VEGF gene delivery improves angiogenesis of senescent endothelial progenitor cells
Source: Sci Rep. 2021 Jun 29;11:13449. doi: 10.1038/s41598-021-92754-3 (PMC8242093; doi:10.1038/s41598-021-92754-3)

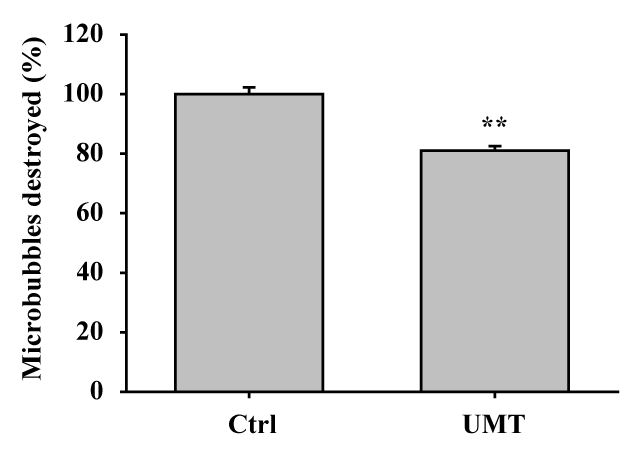

Supplement: Supplementary file 1 — Supplementary Information 1. [file 41598_2021_92754_MOESM1_ESM.tif]

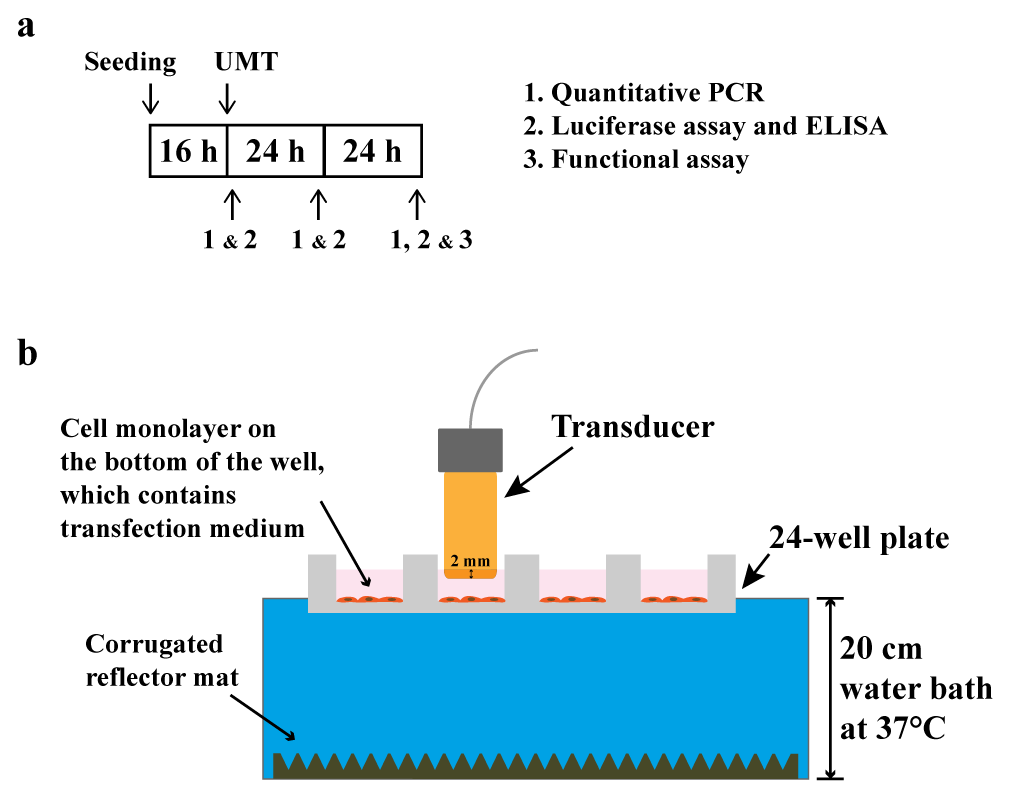

Supplement: Supplementary file 2 — Supplementary Information 2. [file 41598_2021_92754_MOESM2_ESM.tif]

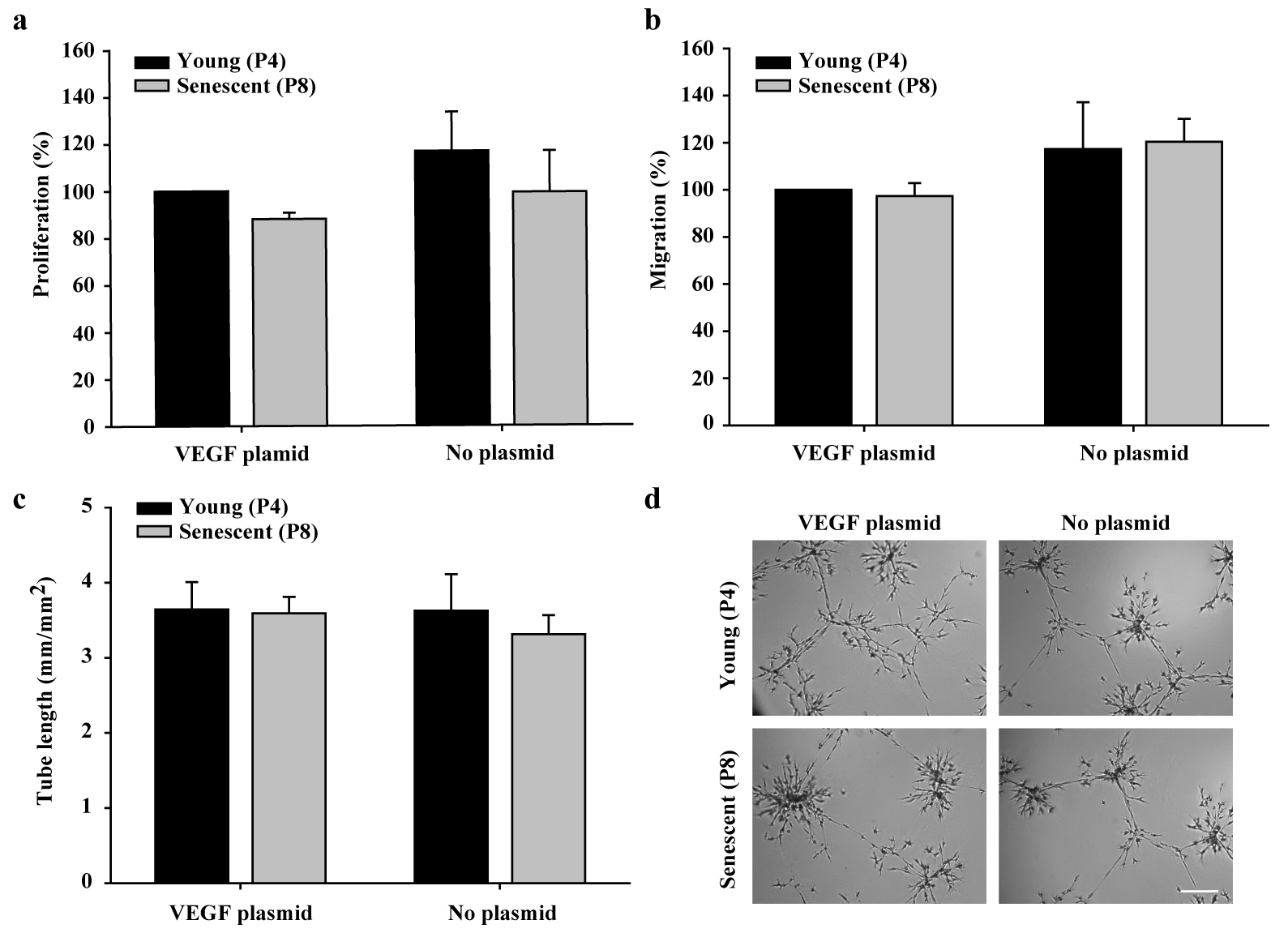

Supplement: Supplementary file 3 — Supplementary Information 3. [file 41598_2021_92754_MOESM3_ESM.tif]
