# Supplementary material for: Ultrasonic microbubble VEGF gene delivery improves angiogenesis of senescent endothelial progenitor cells
Source: Sci Rep. 2021 Jun 29;11:13449. doi: 10.1038/s41598-021-92754-3 (PMC8242093; doi:10.1038/s41598-021-92754-3)
Supplement: Supplementary file 4 — Supplementary Information 4. [file 41598_2021_92754_MOESM4_ESM.docx]

**Supplementary material**

Fig. 1.


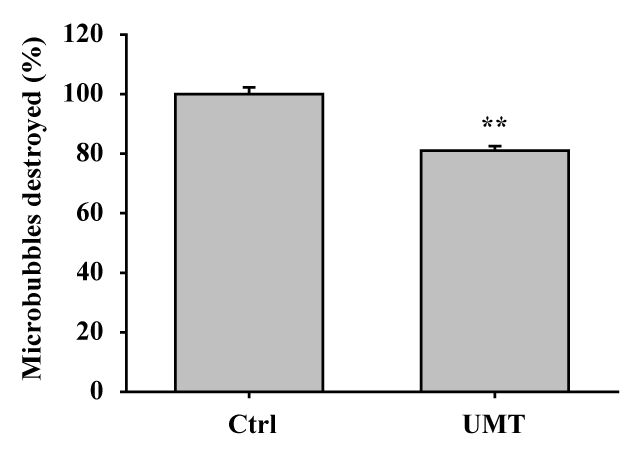


Fig. 1. Percentage of microbubbles destroyed by ultrasonic microbubble transfection (UMT). Approximately 20% of the microbubbles in the well were destroyed after UMT (intensity, 0.5 W/cm^2^; DC, 20%; exposure time, 30 sec). n = 3 for each bar. ^**^, *p*＜0.01 vs. control.

Fig. 2.


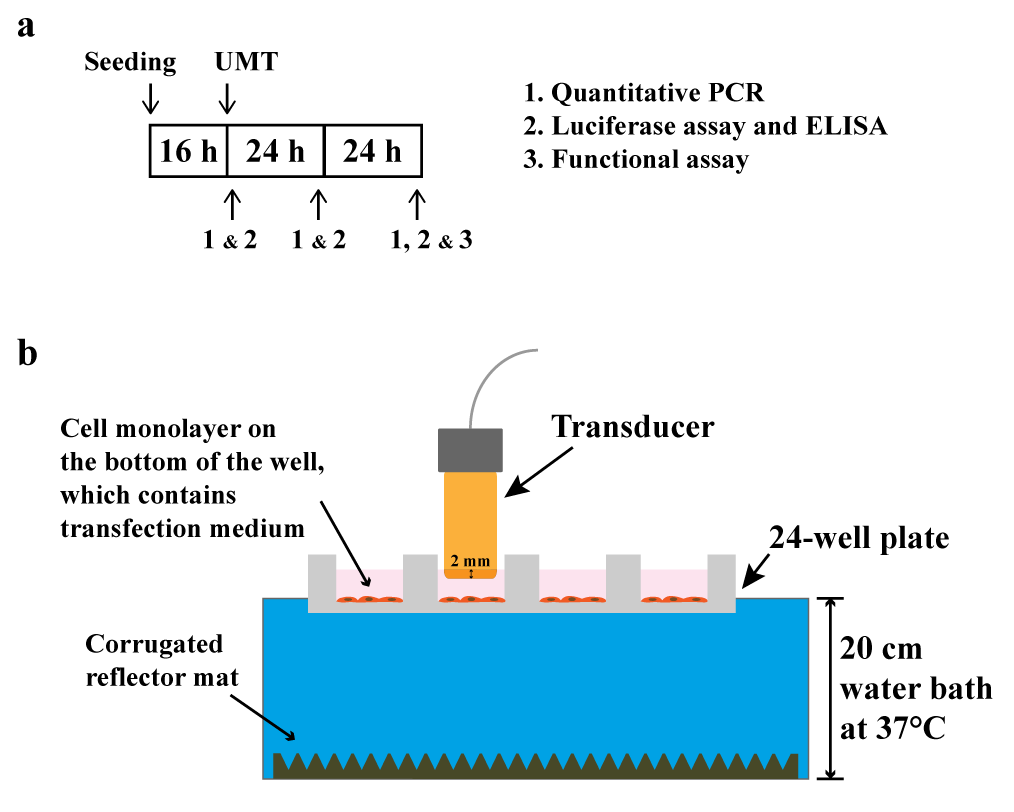


Fig. 2. Illustration of the apparatus used to perform ultrasonic microbubble transfection (UMT). (**a**) The transfection scheme shows that treatments were performed at different time points beginning at 16 hours (h) after cell seeding. (**b**) The ultrasound apparatus schematic shows that a 24-well plate was suspended in a polystyrene water bath at 37 °C during ultrasound exposure. A corrugated silicone mat was fixed to the bottom of the water bath to allow sufficient space for the reflected waves to escape without interfering with the rest of the 24-well plate. The ultrasound probe was slotted directly into the cell suspension in the culture well, with the transducer 2 mm below the surface of the transfection medium. Supplementary figure 2b was created using Adobe Illustrator CC 2018 version 22.0.1.

Fig. 3.


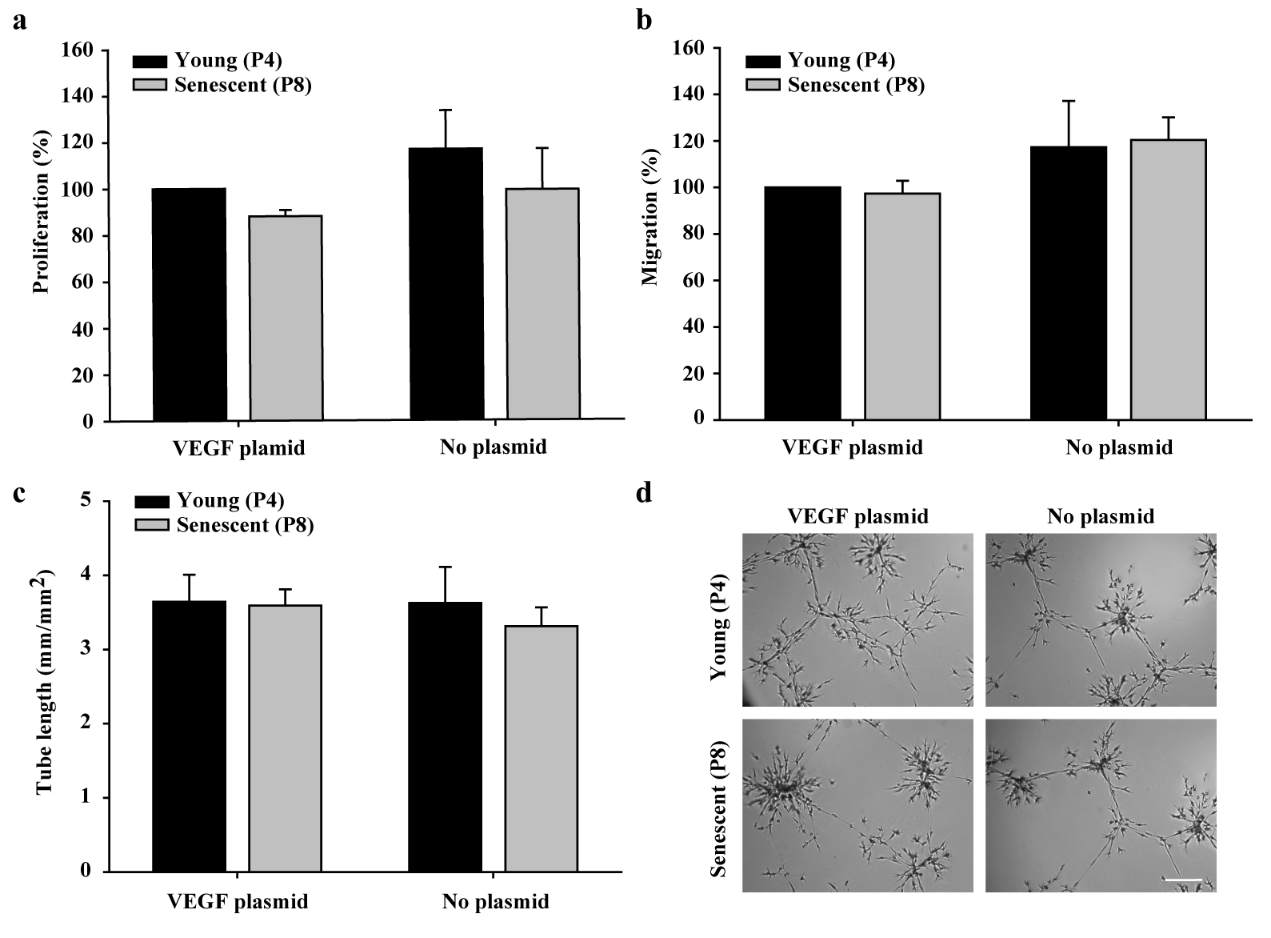


Fig. 3. Evaluation of the effect of VEGF plasmid DNA on the angiogenic properties, proliferation (**a**), migration (**b**) and tube formation (**c**), of young and senescent porcine EPCs. There is no significant difference between each bar of the same group or between the corresponding bars of different groups. (**d**) Representative micrographs of tube formation in the different groups are shown. Scale bar: 250 µm. Migration and tube formation were analysed with Leica QWin image analysis software (Cambridge, UK, version number: V3.5.2). n = 6 for each bar.
